# The Structure and Compression of Medical Compression Stockings

**DOI:** 10.3390/ma15010353

**Published:** 2022-01-04

**Authors:** Miloš Lozo, Željko Penava, Ivo Lovričević, Zlatko Vrljičak

**Affiliations:** 1 Department of Textile Design and Management, Faculty of Textile Technology, University of Zagreb, 10000 Zagreb, Croatia; mlozo@ttf.unizg.hr (M.L.); zvrljicak@ttf.unizg.hr (Z.V.); 2 Department of Surgery, Sestre Milosrdnice, University Hospital Centre, 10000 Zagreb, Croatia; ilovrincevic@mef.unizg.hr

**Keywords:** compression stocking, elastane, elongation, compression

## Abstract

This paper lays out standards of compression stockings and their classification into classes. The analysis of knitted fabric structure parameters, elongation and compression of moderate- and high-compression stockings was conducted. Stocking compression on specific parts of the stocking leg was measured on three sizes of a wooden leg model. For moderate-compression stockings, compression above the ankle was 32 hPa. For high-compression stockings, compression above the ankle was 60 hPa. Both groups of the analyzed compression stockings were made on modern one-cylinder hosiery automats. The legs of the stockings were made in single inlaid jersey 1 + 1. Both yarns were elastane covered. The finer yarn formed loops and its knitting into a course was significantly larger than in the other yarn, which was much coarser and does not form loops but “lay the weft in a bent way”. The smallest elongation of knitted fabric was above the ankle, where the highest compression was achieved, while the largest elongation was under the crotch, where the stocking leg exerted the smallest compression on the surface. The leg of the compression stocking acted as a casing that imposed compression on the leg and often reinforced it to be able to sustain compression loads.

## 1. Introduction

Contemporary humans do not seek only objective value, but also subjective satisfaction in life. Custom-made clothes have been around for a long time [1]. Modern technical and technological development has enabled the personalized production of clothes and hosiery, particularly special purpose or medical (therapeutic) compression stockings [2,3,4]. Special purpose stockings are characterized by a specific construction and material composition. The production of good quality and functional stockings with a special purpose and construction is based on correct measurements of the leg the stockings will be used on [5,6].

During use, some parts of the stocking achieve compression on the leg of up to 67 hPa (67 g/cm^2^ or 50 mmHg, where 1 mmHg = 1.33 hPa) [7,8]. Hosiery compression on the leg, which is usually up to 13 hPa (10 mmHg), is considered small and not stated on hosiery [9]. Thus, for example, the body of a short classic sock achieves compression from 4 to 8 hPa, while the elastic cuff that helps the sock stay up achieves compression ranging from 6 to 13 hPa, which is not stated on the manufacturing label [10,11]. Fine classic women’s stockings also provide a small amount of compression that reaches 8 hPa on certain leg parts. However, stockings that press the leg a little more exert 5 to 15 hPa and are often called high-compression stockings [12,13]. They are usually worn by women on special occasions. People who spend most of their working hours standing, such as surgeons, dentists, waiters, hairdressers and shop assistants, use preventive compression stockings that exert compression on certain leg parts from 5 to 27 hPa (4 to 20 mmHg). These socks help people to perform their daily activities for longer and with less pain and strain, and thereby protect from (prevent) leg diseases [14,15]. Some recreational and professional athletes also use such preventive stockings on various occasions. Compression stockings are an integral part of therapeutic prophylaxis for sick legs. Depending on the disease type, compression stockings may exert compression on a particular leg part of up to 67 hPa (50 mmHg). The strongest compression in medical compression or therapeutic stockings is typically above the ankle and gradually decreases toward the crotch. Depending on the disease and therapy, stocking compression from the ankle to crotch can even decrease by up to 80% [16,17,18].

When measuring stocking compression on the leg, we measure the force with which a stocking presses the surface. In rigid-body mechanics, the ratio of force and surface area on which the force acts is called stress, while in fluid mechanics, the ratio of force and surface area is called pressure [19,20]. Blood pressure is measured in medicine and, in practice, it is still expressed in millimeters of mercury (mmHg). Some designs of medical compression stockings aim to compress the tissue, but primarily blood vessels, in order to decrease their volume and thereby produce an effect on blood pressure. The compression of these stockings is also measured in millimeters of mercury. This was the basis for determining the standards for classes of hosiery compression on the leg, with the compression being expressed in millimeters of mercury. However, in technical sciences, pressure is measured in Pa (pascals), whose unit is newtons per square meter (where 1 N/m^2^ ≈ 100 g/m^2^). The two main units used to measure hosiery compression on the leg are often stated on products or in research results [21,22].

Some developed European countries have their own norms to determine hosiery compression on the leg [7,8,9]. Due to the wide range and quality of applications, compressions are typically divided into four classes or grades. Compressions up to 13 hPa (10 mmHg) are generally not of interest for medical therapeutic purposes and cannot be found in the norms. According to the French norm, the first class includes stockings that exert light compression from 10 to 15 mmHg. In the English norm, the first class includes stockings that achieve compression from 14 to 17 mmHg, and in the German norm, from 18 to 21 mmHg. The compression of the second class in the French norm approximately corresponds to the first class of the German norm, i.e., the compression of the third class in the English norm corresponds to the second class of the German norm.

The given norms are primarily used to produce (therapeutic) medical compression stockings. Doctors use the classes as guidelines to prescribe compression stockings for specific diseases [15,23,24]. However, such divisions create confusion in applications, especially in other countries of the European Union. Stockings that exert compression of up to 20 mmHg (27 hPa) above the ankle are often used without doctor consultation. These stockings are worn by recreational athletes and former professional athletes, as well as pregnant women. An experienced or recreational athlete will adjust the hosiery compression to their activities without consulting a doctor. Many pregnant women will happily consult a doctor on the use of preventive compression hosiery, especially after the second childbirth.

The term stocking compression on the leg means the pressure with which the stocking presses the leg. Pressure can be measured on models (in vitro) or people (in vivo) [16,17,22]. Doctors measure stocking compression on patients in vivo, but in production, research and laboratories, stocking compression is usually measured on a model, i.e., in vitro. When stocking compression is measured in production or laboratories, the whole or part of the stocking is pulled onto rigid cylinders of different diameters to measure the elongation and compression on the surface. Different elongations and compressions are achieved at certain cylinder diameters. In order to imitate the stocking position on the leg, it is mounted on an artificial leg, which is most commonly wooden, and the compression is measured in different places [16,25,26]. When measuring the stocking compression on a person, i.e., on a real leg, measurements are performed in a similar way. In this case, the largest part of the stocking is mounted onto a soft surface and a measuring sensor is inserted between the stocking and leg. The results obtained on a model and the human leg will be different [10,11,16]. The results of measuring stocking compression at different bidirectional elongations or those obtained through different measuring methods will also differ [26,27,28]. In any case, the relevant compression values are those measured on the human leg, and any other results should be compared or adjusted to those. On the free market, there are different constructional shapes of compression stockings that basically have a targeted application. It is not recommended that the end user wears such stockings without consulting a doctor and/or another expert first. Therefore, this study analyzed stockings with moderate (32/11 hPa) or high compression (60/27 hPa) [7]. The focus was on the structural analysis of the knitted fabric used to make stockings, elongation sizes and compression on specific leg parts. Each analyzed compression stocking had certain properties and represented a certain group of compression stockings whose construction is used to produce large batches of different sizes for an unknown buyer on the world market.

The aim of this study was to investigate and analyze knitted fabric structure parameters and the compression of moderate- and high-compression stockings.

## 2. Experimental Part

Two different stockings were used in this experiment: medical moderate-compression pantyhose and medical high-compression pantyhose.

### 2.1. Wooden Model Leg

**Figure 1:**
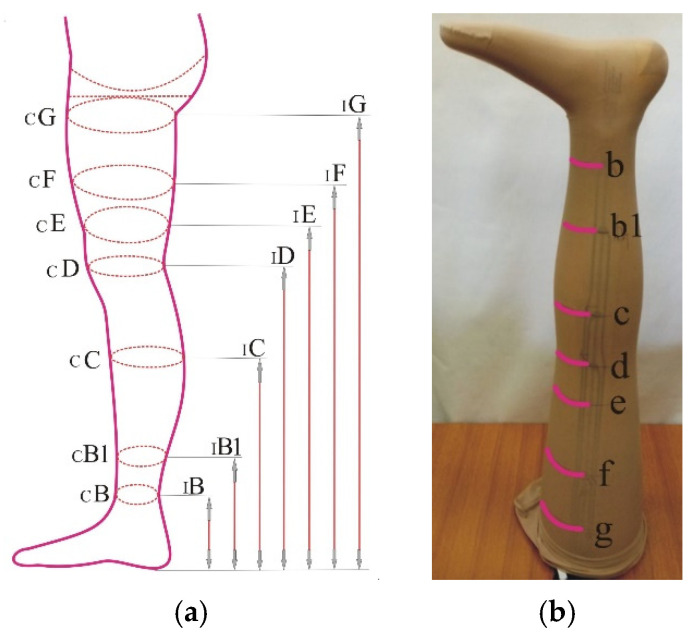
Positions for leg measurements: (**a**) for height and circumference; (**b**) marks on the wooden leg model when measuring compression.

In the analysis of these compression stockings, the focus was on the structure of the stocking leg and its compression on a rigid cylindrical surface. The structures of the foot, panties or stitches are irrelevant for this research. The analysis of the stocking starts by taking measures of specific leg parts (Table 1, Figure 1).

After measuring the widths and heights of certain segments, marks (markers) were put on each stocking for specific points where the compression was measured. Next, each stocking was mounted on the wooden leg model. All stockings were intended for adults and mounted on three sizes of the wooden leg model: M7, M9 and M11 (Table 1).

The height of all three wooden leg models was 80 cm [25]. According to the manufacturer’s request, on the wooden leg models, 7 positions for measuring compression (b, b1, c, d, e, f, g) were marked (Figure 1). The wooden leg model was placed longitudinally on a worktop (table, bench) with the foot facing up (Figure 1b). A strip of compression sensors was attached to the foot of the wooden leg with adhesive tape. The tape with seven measuring points was lowered from the foot to the crotch, whereby the sensors on the tape coincided with the markers on the wooden leg (located at heights of 12, 20, 31, 39, 45, 60 and 72 cm (points I-B to I-G, Table 1).

A stocking as a flat shape was attached to the foot on a special stand. The other side of the stocking that fits the crotch was also fastened with another clamp and the stocking was stretched to a length of 80 cm, which was the length of the wooden leg model. The force gauge was located on this second clamp and the tensile force of the stocking was registered (e.g., 25 N). Then, the stocking was put on the smallest used model of a wooden leg (M7). The stocking was stretched so much that the markings on the stocking coincided with the markings on the wooden leg. Then, the tensile force of the stocking was measured, e.g., 45 N. Therefore, the stretching force of a stocking on a wooden leg was significantly higher than in a flat position. The compression-measuring device was then switched on and the compression was measured at all points at the same time, and the results are displayed on the device screen and can be printed on paper tape. After this fundamental measurement, the stocking was removed from the model of the wooden leg and again after a few minutes, it is put on the leg and, by assessment, positioned to fit the leg comfortably. In this case, the markers on the stocking did not match the markers on the leg. The stretching force of the stocking and the compression at the above points were also measured. After this measurement, the stocking was taken off the leg and pulled on again, and for the third time, the stretching force of the stocking and the compression were measured. In this third pull of the stocking onto the leg, we tried to pull the knitted fabric onto the corresponding part of the leg. Slight deviations in the compression values were obtained for all compression measurements for different combinations and time intervals. This was due to the different lengths of stretching knitted fabrics on individual parts of the stocking. In this way, we imitated the position of the stocking in use on the human leg. Stockings never stand in the same place on a human leg, especially when walking for a long time. This measurement method was also applied when measuring the compression on the other two larger legs, i.e., models M9 and M11. As these were compression medical stockings, the measurements were repeated several times at different time intervals.

### 2.2. Characteristic Parameters of Yarns

**Figure 2:**
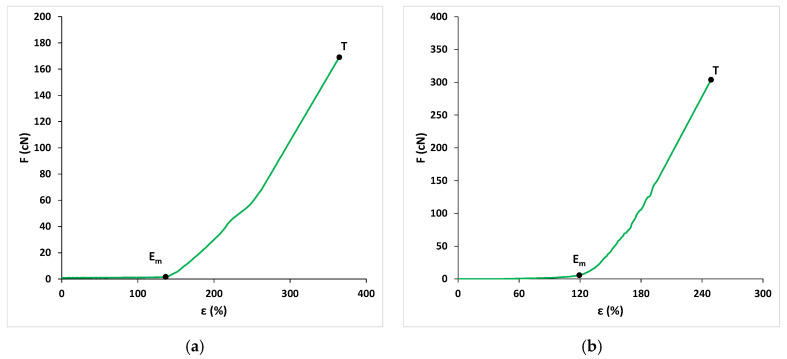
Force–elongation diagrams for the double-covered elastane yarns used to make compression stockings, measured without preload: (**a**) nominal yarn count of 52 dtex for the yarn used to make loops; (**b**) nominal yarn count of 232 dtex for the yarn that did not make loops but “lay the weft in a bent way”.

**Figure 3:**
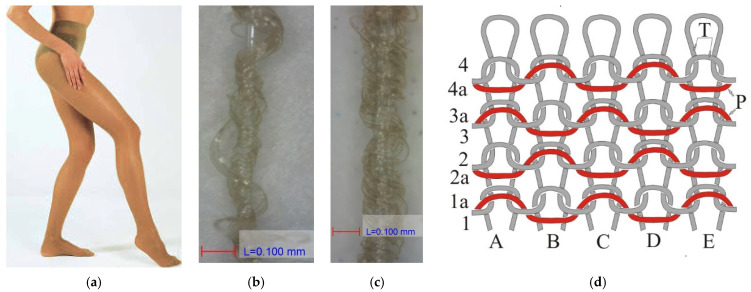
Yarns and structure of knitted fabric in women’s moderate-compression pantyhose: (**a**) the appearance of pantyhose; (**b**) double-covered elastane yarn with a nominal count of 52 dtex, which was used to make loops; (**c**) double-covered elastane yarn with a nominal count of 232 dtex, which was not used to make loops but to “lay the weft in a bent way” in rapport 1 + 1; (**d**) structure, jersey, single inlaid 1 + 1 used to make the largest part of the stocking; *T*—ground yarn, P—“bent weft“.

Two types of yarn were used to make the pantyhose for this experiment. Both yarns were double-covered elastane. The point *T* (ε, F) represents the breaking elongation and the corresponding value of the force at break. Its breaking elongation, measured without preload, was 364 ± 43%, and the largest force registered at break was 169 ± 14 cN. The force–elongation diagram for a nominal yarn count of 52 dtex is shown in Figure 2a. Elastic deformation of this yarn (ε_m_) was 140% or 38% of the total elongation. For such an elongation, it is necessary to have a force of 2 cN, or around 1% of the largest force measured at break (point E_m_). The second yarn, which did not make loops, had a breaking elongation of 251 ± 28% without preload and the largest force at break of 311 ± 22 cN. The force–elongation diagram for a nominal yarn count of 232 dtex is shown in Figure 2b. The amount of elastic deformation in this yarn was also large and it amounted to 120%, while the measured force was 3.2 cN, i.e., also 1% of the breaking force. Both yarns obviously had a large elastic deformation (>100%). Therefore, when being knitted in the stretched state, they simply intertwined, while the yarn that did not make loops lay around two neighboring loops with a bent weft appearance. Therefore, such samples are often described as weft-knitted fabric. The first yarn was finer, with an average total nominal count of 52 dtex; it made smooth loops (*T*) on each needle (Figure 3b).

The second yarn was significantly coarser, with an average nominal count of 232 dtex; it was not used to make loops but laid the weft (P) in a special way in the laying rapport 1 + 1 (Figure 3c). These two yarns were intertwined in the leg of the stocking in the classic plain inlaid structure 1 + 1.

Of the four yarns listed, three were double-covered elastane yarns. The core was elastane and the cover was nylon—PA 6.6. The proportions were about 70% elastane + 30% PA 6.6. In the case of single-covered yarn, the relationship is reversed, i.e., about 30% elastane + 70% PA 6.6.

### 2.3. Characteristic Parameters of Medical Moderate Compression Pantyhose

In the German norm, stockings belong to class II if they exert moderate compression on the leg [7]. They are recommended for varicose veins during pregnancy or in advanced primary varicoses with edema, thrombophlebitis with accompanying edema, chronic venous insufficiency, etc. These compression stockings have an entire foot, a heel shaped via knitting and panties. They are made on a more advanced construction of a hosiery automat that knits with 420 needles and has the capacity to form a course with two very different yarns (Figure 3).

The compression that the pantyhose legs achieved above the ankle was 32 hPa (24 mmHg) and 11 hPa (8 mmHg) in the crotch area. Characteristic parameters for women’s medical moderate-compression pantyhose are presented in Table 2.

The stocking circumference above the ankle (Table 2, point b) was 15 cm (S_p_ = 150 mm), gradually increasing toward the crotch (point g), where it was 22 cm (S_p_ = 220 mm). The heights of certain stocking widths (widths at specific points) were different, and the total height of the stocking leg was 45 cm. The height of a particular stocking part was at least 5 cm. However, when two neighboring widths were significantly different, the height of the joining part was significantly shorter.

L_oc_—length of elastane yarn knitted into a course to make loops (mm); ℓ_oc_—average length of elastane yarn spent to make a single loop (mm); m_oc_—mass of elastane yarn knitted into a course when making loops, as determined by weighing (g); L_po_—knitting of double-covered elastane yarn into a course, where this yarn did not make loops but laid the weft in a bent way (mm); ℓ_po_—average length of spent double-covered elastane yarn that did not make loops but laid the weft over a ground loop (mm); m_po_—mass of double-covered elastane yarn knitted into a course, as determined by weighing (g); T_toc_—count of elastane yarn used to make loops (dtex); T_tpo_—count of double-covered elastane yarn not used to make loops (dtex); m_e_—mass per unit area of knitted fabric determined on a specific part of the stocking (g/m^2^); S_p_—circumference of tubular knitted fabric on a specific part of the stocking (mm); ε_pl_—elongation of tubular knitted fabric on a specific part of the stocking (%).

The following standards were used to determine the structural parameters of knitted fabrics for stockings and to determine breaking force and elongation:

- ISO 2062:2003: Textiles—Yarn from packages. Determination of single-end breaking force and elongation at break.
- ASTM D8007-15 (2019) was used to determine the wale and course counts of weft-knitted fabrics per unit of length.
- ISO 3801:1977. Determination of mass per unit length and mass per unit area.
- ISO 7211-5:1984. Woven fabrics—construction—methods of analysis—part 5: determination of linear density of yarn removed from the fabric.

### 2.4. Characteristic Parameters of Medical High-Compression Pantyhose

The analyzed stockings were finished, refined and offered on the market. They also had a whole foot and a shaped heel; the compression measured above the ankle was 60 hPa (45 mmHg) and 27 hPa (20 mmHg) under the crotch (Table 3). In the German norm, they are classified as class III compression hosiery, which signifies high compression, and class IV in the French norm, which stands for very high compression hosiery. The stockings were analyzed according to the same principles as the previous ones. The characteristic parameters for women’s medical high-compression pantyhose are presented in Table 3.

This medical high-compression pantyhose was also made on a small specially constructed single-bed circular knitting machine called a hosiery automat. Based on the analyzed parameters of the knitted fabric structure in specific parts of the stocking leg, the estimated diameter of the cylindrical bed of the hosiery automat used to make these stockings was 120 mm (4¾ inch), it knitted with 420 needles and had the count E28. To make a stocking with a smaller elongation and larger compression, ground yarn with a total count of 120 dtex was used to make smooth loops in each course.

**Figure 4:**
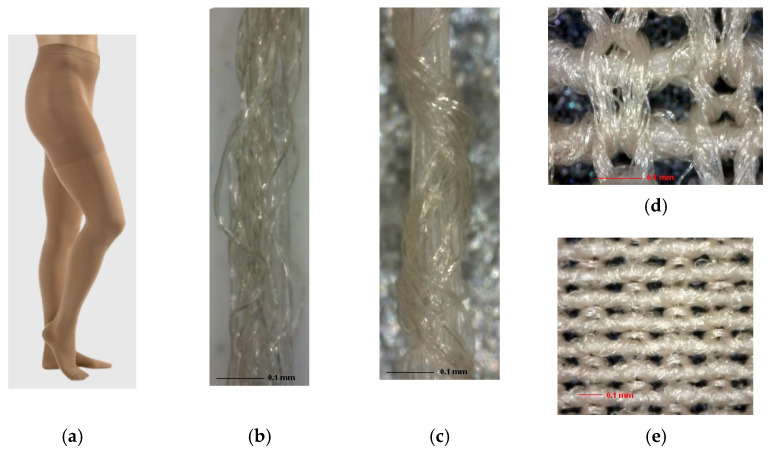
Medical compression stockings with the foot, heel and panties with a high compression of 60 hPa (45 mmHg) above the ankle: (**a**) the appearance of the pantyhose; (**b**) ground single-covered elastane yarn with a total count of 120 dtex, which was used to make loops in each course; (**c**) double-covered elastane yarn with a total count of 700 dtex, which laid the weft in a bent way and exerted high compression; (**d**) front side of the knitted fabric; (**e**) backside of the knitted fabric.

Apart from this yarn, almost six times coarser yarn was knitted into each course, which had a total count of 700 dtex. This yarn laid the weft in a bent way, forming a plain inlaid structure 1 + 1 together with the ground yarn. The second, much coarser yarn, which did not form loops but laid the weft in a bent way, had a significant impact on producing larger stocking compression on the leg (Figure 4).

The total length of the stocking leg was 48 cm, and together with panties, the length was 76 cm. The circumferences of the leg were as follows: the smallest above the ankle (position b) was 170 mm, at the calf (position c) was 220 mm, around the knee was 230 mm, in the middle of thigh muscles (position f) was 330 mm and under the crotch was 340 mm. As the width of the knitted fabric increased, its shrinkage upon its removal from the machine decreased, and its mass per unit area decreased from 298 to 260 g/m^2^. According to the data obtained in the knitted fabric analysis, it could be concluded that the machine operated under almost extreme constructional conditions since the needle bed diameter of 380 mm was used to make tubular knitted fabric that fit around the crotch and had a circumference of 340 mm (shrinkage on the machine around 10%), and only 170 mm above the ankle (knitted fabric shrinkage on the machine of 55%). The smallest length of the knitted ground yarn (L_oc_) with a count of 120 dtex making loops in the area above the ankle was 1000 mm, the knitted length was larger around the knee at 1208 mm and the largest was under the crotch at 1332 mm, i.e., the average length of yarn making a loop (ℓ_oc_) was 2.38 to 3.17 mm.

The masses of ground yarn knitted into a course (m_oc_) also increased from the ankle toward the crotch and were 0.0118 to 0.0161 g. The increase in length knitted into a course was around 33%, and regarding the mass, the increase was 36%. This discrepancy pointed to the change in knitting conditions along the stocking leg, primarily the amount of elongation force when the ground yarn was fed into the knitting zone. The second yarn was not used to make loops but to lay the weft in a bent way (L_po_) and its knitted length was significantly smaller: 180 to 370 mm per course, or 0.43 to 0.88 mm in the position of one ground loop. The mass of the second knitted yarn was larger than the mass of the ground yarn since the second yarn was much coarser. The mass of the second knitted yarn (m_po_) per course was 0.0120 to 0.0271 g, while 0.0238 to 0.0432 g was simultaneously knitted into a course.

## 3. Results and Discussion

### 3.1. For Medical Moderate-Compression Pantyhose

The leg height for this stocking was 45 cm; it was stretched to 80 cm or 78% and the elongation force was 40 N. There were around 1200 loop courses of different densities along the leg height. When stretching the leg on the wooden surface, it was noticeable that certain parts stretched differently and tended to occupy a position that was different from the one marked on the stocking. When the stocking was freely mounted on the wooden leg for the second, third or fourth time and stretched while appropriately fitting on the leg, different compression data were obtained compared to measuring the base compression with adjusted marks. The measured compression even deviated by up to ±2 mmHg. This is very important information that indicates that the compression on the leg will also deviate from the predicted value since the leg will not always be pressed by the same part of the stocking. Elongation of knitted fabric on a specific leg part depends on the amount of elongation force, but also the length of the yarns knitted into a course. This means that there should be a maximum of three yarn widths in the calf area. However, such a stocking would be uncomfortable to use. Therefore, many designers make the stocking calf using more widths that do not fit on particular leg parts according to the predicted distribution; therefore, they exert a different compression.

The measured knitted fabric elongation (ε_pl_) above the ankle (point b) in all the wooden leg models was 47 to 73%, and in the crotch area (point g), the elongation was 127 to 164%; on the wooden leg model M7, the elongation increased from the ankle toward the crotch by 47 to 127%, and the knitted fabric compression (K_c_) on the leg decreased from 31 to 11 hPa, i.e., it fell by 65% (Table 2). The elongation on the larger wooden leg models (M9 and M11) was higher but the knitted fabric compression on the leg did not significantly change. These data suggested that the knitted fabric elongation in a specific leg part occurred in the elastic area where Hooke’s law applies such that a large elongation of knitted fabric was achieved with a small increase in force. Therefore, a legitimate question is: What kind of human leg (i.e., which measurements) is this compression stocking intended for? To provide an answer, all the compressions in the given parts of the stocking and other wooden leg models must be analyzed in more detail. However, this will still not be sufficient to obtain an adequate answer, which is to be sought in the subjective user experience. Furthermore, such compression stockings can also be used on shorter legs with larger circumferences where the achieved compression will range from 34 to 49 mmHg, and the stockings will belong to the high-compression group. It is important to note that the compression of such stockings was around 20% higher at the beginning of the leg calf (position b1) than above the ankle (position b), and only then did it gradually decrease toward the crotch to 24, then 19, 13 and 11 hPa.

Further in the analysis, the leg of the stocking was cut in certain parts from the ankle to the crotch with the aim of determining the length (L) and mass (m) of yarn knitted into a course. This is a very demanding task in the analysis of knitted fabric structure, even for an experienced analyst, since the knitted fabric in question is a very compact elastic knitted fabric made with two double-covered yarns of significantly different counts and breaking elongations. First, the foot of the stocking was cut above the ankle in position b, and then the tubularly shaped knitted fabric (leg) was mounted on the wooden leg model. This part had the smallest circumference of knitted fabric, which made it easy to count the loops in a course. There were 420 loops in a course, i.e., the machine knitted with 420 needles. Next, the tubular knitted fabric was cut longitudinally, partially between two wales, and was torn out from a whole course yarn by yarn. A single course was formed by two yarns. Both were double-covered elastane. First to be torn was the yarn that formed loops, followed by the yarn that did not form loops but “laid the weft in a bent way”. The yarns torn from ten courses were laid in order in two groups on a flat surface. The yarns were obviously in different and unknown counts. The finer yarn that formed loops had large wavy deformations, while the coarser yarn, which did not form loops but laid the weft in a bent way, had very small deformations. To determine the length of yarn torn from a course, it is necessary to have a preload, which depends on the yarn count. However, the yarn count was unknown, meaning the adequate preload could not be applied. It is very hard to estimate the yarn count of double-covered elastane, especially fine yarn that could be used to determine the preload. Based on different trial measurements, a preload that flattened the yarn deformation that occurred due to knitting and refining of the stocking was accepted. For the ground yarn that made loops, the applied preload was 1.5 g, whereas, for the yarn that did not make loops but laid the weft in a bent way, a preload of 0.5 g was applied. The entire leg of the stocking was made using these two yarns so that the given preloads were applied at all places where the yarn was torn out from the knitted fabric from the ankle (position b) to the crotch (position g).

**Figure 5:**
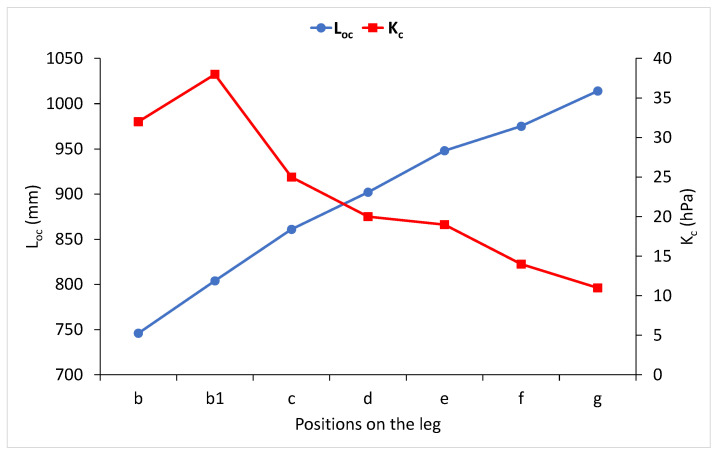
The relation of the length of the ground yarn knitted into a course (L_oc_) and the compression (K_c_) at a specific position on the wooden leg model of size M9 for moderate compression.

With the given preloads, the average length of the knitted ground yarn that made loops in a course (L_oc_) in the area above the ankle (position b) was 746 mm, above the calf (position c) was 861 mm, in the middle of the thigh (position f) was 975 mm and at the crotch (position g) was 1014 mm. In the area that fit around the base of the calf (position b1) and in the knee area, i.e., the d and e areas, the length of knitted yarn varied every few courses and is therefore not given. When the given amounts were divided by the number of needles that participated in the knitting, it could be noticed that the average length that was knitted to make one loop (ℓ_oc_) was 1.78 to 2.41 mm. The knitted length of the second yarn, which did not form loops but laid the weft in a bent way in a course (L_po_), was significantly smaller, i.e., 195 to 395 mm, or in the area of one ground loop (ℓ_po_), the knitted length was 0.46 to 0.94 mm on average. These data pointed to the particularity of such a knitted fabric structure and the demanding task of its analysis. All the torn yarns that had the same count and measured length were put together on a scale where the mass of the yarns knitted into a course was measured simply and precisely. The average mass of the ground yarn knitted into a course (m_oc_) was 0.0036 to 0.0057 g, and 0.0043 to 0.0096 g for the yarn that did not form loops (m_po_), i.e., 0.0079 to 0.0153 g was knitted into one course. There was a strong mathematical correlation between the precisely measured mass of yarn knitted into a course and the compression (Figure 5).

The average nominal yarn count was determined based on the measured length and mass. The ground yarn that made loops had a count of 52 dtex, and the yarn that did not make loops had a count of 232 dtex (Table 2, column e). This research did not aim to establish the real count of particular yarns since to arrive at these data, it would be required to conduct a special and long analysis that would involve determining the number and count of filaments in the core and both coverings, the number of coils and, finally, the real yarn count.

The results obtained by measuring the compression in moderate-compression stockings on specific parts of the wooden leg model are shown in Table 4.

The mass per area of knitted fabric in a specific stocking part was determined before measuring the length of yarns knitted into a course. The stocking part that fit around a specific leg part was cut out from the stocking leg. Its width, height and mass were measured. The mass per area of knitted fabric in a specific part of the stocking was determined based on the mass and area of the knitted fabric which was cut out: the area mass around the ankle (position b) was 168 g/m^2^, and under the crotch (position g), it was 161 g/m^2^. In this way, both stocking legs, or one pantyhose sample, were destroyed during the analysis.

Based on the total analysis of knitted fabric structure in the stocking leg, it could be concluded that the smallest width of knitted fabric was obtained above the ankle, whereas the smallest length of yarn knitted into a course was obtained at the smallest sinking depth and the largest elongation force when the yarn was fed using needles. This knitted fabric structure stretched the least and, at the same time, achieved the largest compression of knitted fabric on a rigid cylindrical surface. The largest width of knitted fabric was measured at the top of the stocking leg fitting under the crotch. This width was obtained using a much larger length of yarn knitted into a course, which was achieved at a much larger sinking depth and probably a smaller elongation force when feeding using needles. These are basic parameters linking the width of tubular knitted fabric, its elongation and the achieved compression. These data can be the basis for designing and planning the production of similar compressive pantyhoses.

### 3.2. For Medical High-Compression Pantyhose

When this type of stocking was mounted on the three wooden leg models, it stretched from 48 to 80 cm or around 67% longitudinally, after which, the compression was measured. The elongation forces of the leg on the three sizes of the wooden leg model were 45, 47 and 50 N. Such biaxial (bidirectional) stress caused a completely different compression compared to the uniaxial (unidirectional) or transversal elongation. The compression measured on the wooden legs was the largest in the area above the ankle (position b) and at the beginning of the calf (position b1) at around 60 hPa (45 mmHg). As in many medical compression stockings, the compression gradually decreased from the ankle toward the crotch (Table 5). Above the knee (position d), the compression decreased 52 to 48%, in the area under the crotch, it decreased 59 to 55%, and amounted to 24 to 27 hPa, i.e., 41 to 45% of compression above the ankle. It was obvious that high compression dominated throughout the entire stocking leg. From a medical therapeutic standpoint, the stockings are used in prominent chronic venous insufficiency, thrombophlebitis, posttraumatic edema, post-thrombotic edema or reversible lymphedema.

The results obtained by measuring the compression of high-compression stockings on specific parts of the wooden leg model are shown in Table 5.

In tubular products of this kind, it is interesting to analyze the elongation of tubular knitted fabric (ε_pl_) and the measured compression (K_c_). The body of the stocking was made with different circumferences and widths (S_p_), and each width stretched differently to achieve the given compression. As in the previous analysis, the stockings were mounted on the wooden leg model in three sizes (M7, M9 and M11), whose circumferences above the ankle were 22, 24 and 26 cm, respectively.

**Figure 6:**
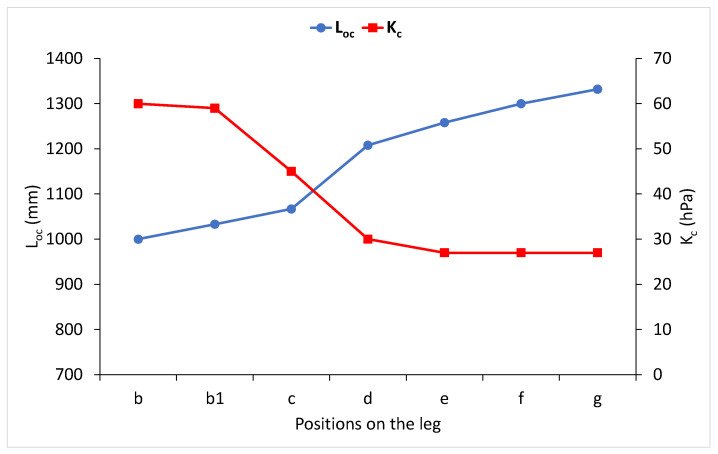
The relation of the length of the ground yarn knitted into a course (L_oc_) and the average compression (K_c_) at specific parts of the wooden leg model of size M9 for high compression.

When a stocking was mounted on three sizes of the wooden leg model, it stretched the least above the ankle while the measured compression was the highest. The elongation was 29 to 53%, and the compression was 58 to 61 hPa. The biggest compression of 57 to 84% was achieved on the calf (position c), while the compression decreased by 23 to 26% in relation to the compression above the ankle (position b) and was 43 to 47 hPa. A smaller stocking elongation of 16 to 18% was measured in the crotch area (position g) than in the calf, and the smallest compression was also measured here: 24 to 27 hPa. According to the conducted analysis, it could be concluded that in this stocking, there was no firm mathematical correlation between the amount of elongation and the compression of the knitted fabric. It can be suggested that knitted fabric elongation occurred in the elastic area, where a small increase in force caused a large elongation, which is not shown in the measured compression. Therefore, the same question can be posed here: What leg size is this compression stocking intended for? However, it is obvious that as the length of ground yarn knitted into course increased, no matter the errors made when it was determined, the average compression decreased from 60 to 26 hPa, and it can be described using a simple mathematical correlation (Figure 6).

## 4. Conclusions

The classic construction of a medical (therapeutic) compression stocking follows the principle of the highest compression in the ankle area, which gradually falls toward the crotch. Depending on the purpose, it is possible to produce a compression stocking with assigned amounts of compression in specific leg parts. The right construction of a compression stocking is achieved by adjusting for a few basic parameters.

1. It is important to know the purpose of the stocking, e.g., if it is intended for someone who is healthy or sick, mobile or immobile or if the stocking is used in an air-conditioned, cold or hot area/space.
2. According to the purpose of the stocking, it is necessary to choose a machine of certain construction, fineness and needle bed diameter (length) that will be used to produce the stocking. The knitting technologist who designs the stocking structure must be given a sketch of the leg shape (pictogram) that contains leg lengths and circumferences at specific main points and the desired compression on each specific leg part. In modern production, compression stockings are made according to the personalization principle.
3. The count and structure of the elastane yarns used to produce stockings are chosen based on the obtained input data. A certain width of knitted fabric in a specific part of the stocking (leg of the stocking) that is to exert the required compression at certain elongation is achieved by regulating the sinking depth and elongation force when the yarn is fed into the knitting zone. Elongation properties achieved by knitting must remain largely unaffected by the refinement process.
4. The structure of the knitted fabric and the stretching and compression values of the stocking were analyzed in this study. The analyzed stocking whose compression above the ankle was about 32 hPa and 11 hPa below the groin was a stocking of moderate compression. According to the German norm, this stocking belongs to class II. The second analyzed stocking achieved high compression above the ankle (class III), which was about 60 hPa and 27 hPa below the groin. These stockings are usually intended for the treatment of various venous diseases.

## Figures and Tables

**Table 1:** Wooden leg model measurements for measuring the stocking compression [25].

Leg Sizes		4	5	6	7	8	9	10	11	12	13	14
Circumference (cm)	g_max_	53	55	57.5	60	62.5	65	67	69.5	72	74.5	77
	f_max_	48	50	51.5	53	55	57	58.5	60	62	64	65.5
	g	44	46	48	50	52	54	56	58	60	62	64
	f	41	42.5	44	45.5	47	48.5	50	51.5	53	54.5	56
	e	31	32.5	34	35.5	37	38.5	40	41.5	43	44.5	46
	d	28	29.5	31	32.5	34	35.5	37	38.5	40	41.5	43
	c	30	31.5	33	34.5	36	37.5	39	40.5	42	43.5	45
	b1	24	25.5	26.5	27.5	29	30	31.5	32.5	34	35	36.5
	h	28	29	30	31	32	33	34	35	36	37	38
	b-b	19	20	21	22	23	24	25	26	27	28	29
Height (cm)	I-B	12										
	I-B1	20										
	I-C	31										
	I-D	39										
	I-E	45										
	I-F	60										
	I-G	72										

**Table 2:** Analysis parameters for women’s medical moderate compression pantyhose.

Medical Moderate Compression Pantyhose; “Weft” in Each Course with Laying Rapport 1 + 1; 420 Wales								
Parameter		Points on the Wooden Leg Marked to Measure Certain Parameters						
		b	b1	c	d	e	f	g
L_oc_ (mm)		746	804	861	902	948	975	1014
ℓ_oc_ (mm)		1.78	1.91	2.05	2.15	2.26	2.32	2.41
m_oc_ (g)		0.0036	0.0040	0.0043	0.0047	0.0051	0.0054	0.0057
L_po_ (mm)		195	223	250	292	323	358	395
ℓ_po_ (mm)		0.46	0.53	0.60	0.70	0.77	0.85	0.94
m_po_ (g)		0.0043	0.005	0.0057	0.0063	0.0075	0.0086	0.0096
T_toc_ (dtex)		48	49	50	52	52	55	56
T_tpo_ (dtex)		218	224	228	216	232	241	243
m_e_ (g/m^2^)		168	167	166	168	169	170	161
S_p_ (mm)		150	164	176	184	194	204	220
ε_pl_ (%)	M7	47	72	96	105	114	123	127
	M9	60	87	113	121	129	138	145
	M11	73	102	130	137	144	152	164

**Table 3:** Analysis parameters for women’s medical high-compression pantyhose.

Medical High Compression Pantyhose; “Weft“ in Each Course with Laying Rapport 1 + 1; 420 Wales								
Parameter		Points on the Wooden Leg Marked to Measure Certain Parameters						
		b	b1	c	d	e	f	g
L_oc_ (mm)		1000	1033	1067	1208	1258	1300	1332
ℓ_oc_ (mm)		2.38	2.46	2.54	2.88	2.99	3.10	3.17
m_oc_ (g)		0.0118	0.0122	0.0126	0.0147	0.0153	0.0159	0.0161
L_po_ (mm)		180	200	220	240	290	340	370
ℓ_po_ (mm)		0.43	0.48	0.52	0.57	0.69	0.81	0.88
m_po_ (g)		0.0120	0.0137	0.0153	0.0165	0.0204	0.0243	0.0271
T_toc_ (dtex)		118	118	118	121	120	122	121
T_tpo_ (dtex)		668	683	696	685	700	715	732
m_e_ (g/m^2^)		298	293	288	278	275	272	260
S_p_ (mm)		170	196	220	230	280	330	340
ε_pl_ (%)	M7	29	29	57	41	40	38	47
	M9	41	43	70	54	51	47	59
	M11	53	69	84	67	62	56	71

**Table 4:** Compressions of moderate-compression stockings measured on specific parts of the wooden leg model.

Compression	Size of the Leg Model	Positions for Measuring Compression on the Wooden Leg Model						
		b	b1	c	d	e	f	g
Measured compression (hPa)	M7	31	37	24	19	19	13	11
	M9	32	38	25	20	19	14	11
	M11	33	40	27	20	20	15	12

**Table 5:** Compressions of high-compression stockings measured on specific parts of the wooden leg model.

Compression	Size of the Leg Model	Positions for Measuring Compression on the Wooden Leg Model						
		b	b1	c	d	e	f	g
Measured compression (hPa)	M7	58	56	43	28	27	24	24
	M9	60	59	45	30	27	27	27
	M11	61	60	47	32	29	27	27

## Data Availability

Not applicable.
